# Astragalus polysaccharide promotes the release of mature granulocytes through the L-selectin signaling pathway

**DOI:** 10.1186/s13020-015-0043-z

**Published:** 2015-07-03

**Authors:** Ping-Ping Zhang, Zhao-Ting Meng, Liu-Chun Wang, Lei-Ming Guo, Kai Li

**Affiliations:** Department of Thoracic Medical Oncology, Lung Cancer Diagnosis and Treatment Center, Tianjin Medical University Cancer Institute and Hospital, National Clinical Research Center for Cancer; Key Laboratory of Cancer Prevention and Therapy, Tianjin, 300060 China; Department of Radiotherapy, Hubei Cancer Hospital, Wuhan, 430079 China; Tianjin Institute for Biomedicinal Research, Tianjin, 300050 China

## Abstract

**Background:**

This study aims to investigate the leukogenic effect of astragalus polysaccharide (APS), to compare its effect of increasing the numbers of mature granulocytes with that of granulocyte colony-stimulating factor (G-CSF), and to investigate the mechanism.

**Methods:**

Rats were arbitrarily grouped into four groups (control, cyclophosphamide (CTX), CTX + APS, and CTX + G-CSF groups), and each group was then arbitrarily divided into five subgroups according to the time period since CTX infusion (0, 4, 7, 10, and 14 days). The expression of leukocyte selectin (L-selectin), its ligand, and shedding-related protease on granulocytes was analyzed. Leukocyte counts were obtained. Chemotactic capacity of polymorphonuclear leukocytes (PMNLs) was assessed.

**Results:**

Both APS and G-CSF restored the expression of L-selectin, P-selectin glycoprotein ligand-1 (PSGL-1), CD11b/CD18, and ADAM17 to normal levels (*P* > 0.05 *vs.* control group on each time point), with APS eliciting a greater effect than G-CSF (*P* = 0.005 on day 7, *P* < 0.001 on day 10 and 14 for L-selectin; *P* = 0.038 on day 7, *P* = 0.001 on day 10, *P* < 0.001 on day 14 for PSGL-1; *P* < 0.001 on day 7, 10 and 14 for ADAM17; *P* < 0.001 on day 7, 10, and 14 for CD11b/CD18). The percentages of the bands and segmented bone marrow (BM) cells in myeloid neutrophils were higher in the CTX + APS group than in the CTX group on day 7 (*P* = 0.030) and reached normal levels on day 10 (*P* = 0.547) and 14 (*P* = 0.431) *vs.* control group. The ability of APS to increase numbers of PMNLs in peripheral blood after chemotherapy was significantly superior to that of G-CSF 7 days after chemotherapy (*P* = 0.029 on day 10, *P* = 0.006 on day 14). Moreover, APS more significantly improved the chemotactic ability of PMNLs among mature BM granulocytes and peripheral blood neutrophils after chemotherapy than did G-CSF (*P* < 0.001 on day 7, *P* = 0.001 on day 10 and *P* = 0.005 on day 14).

**Conclusions:**

APS promoted the differentiation and chemotactic ability of BM granulocytes *via* the L-selectin signaling pathway.

**Electronic supplementary material:**

The online version of this article (doi:10.1186/s13020-015-0043-z) contains supplementary material, which is available to authorized users.

## Background

Chemotherapy frequently causes neutropenia, which is a common and severe adverse effect that interrupts the treatment. Granulocyte colony-stimulating factor (G-CSF) is used to promote the proliferation of bone marrow (BM) hematopoietic cells as a treatment for neutropenia. However, apoptosis of hematopoietic progenitor cells and increased proliferation split times become evident after G-CSF stimulation [1]. Moreover, BM does not have enough niches for the implantation of proliferated hematopoietic stem/progenitor cells; as a result, these cells may migrate directly to the peripheral blood and undergo apoptosis in the liver, spleen, and lung, causing a transient increase of leukocytes in blood [2]. Furthermore, long-term and excessive administration of a stimulating factor can lead to increased expression of matrix metalloproteinase-9 (MMP-9) in CD34+ cells (naive hematopoietic cells), which dissolves the BM sinusoid matrix, thereby allowing proliferated hematopoietic stem/progenitor cells to move into the peripheral blood [3]. The deformation, movement, and phagocytic abilities of CD34+ cells are weaker than those of mature cells, so the presence of these cells in peripheral blood results in a loss of anti-infection activities. Excessive release of naive cells can increase the incidence of leukemia [3]. The long-term administration of stimulating factors may galvanize tumor cell invasion and angiogenesis [4].

In some young patients who undergo first-line chemotherapy, active myeloproliferation at 7 days after chemotherapy indicates that hematopoietic cells are not completely suppressed or that they recover (Additional file 1). Chemotherapy could hinder cell adhesion to the sinusoid epithelium, prevent release of BM polymorphonuclear leukocytes (PMNLs), and change the morphology and number of BM sinusoids [5]. Therefore, failure of BM PMNLs release may also cause neutropenia.

We hypothesized that the interaction between leukocytes and vascular endothelial cells (ECs) when PMNLs were released from BM was similar to the interactions involved in the movement of blood PMNLs out of the capillaries. In such interactions between leukocytes and ECs, as selectin family member (*i.e.*, leukocyte selectin; (L-selectin)) [6] is involved in the initial attachment of circulating leukocytes to the endothelium. P-selectin glycoprotein ligand-1 (PSGL-1) on adherent neutrophils acts as a functional ligand for L-selectin [7]. Afterwards, L-selectin is rapidly shed owing to the action of ADAM17, a proteolytic enzyme [8]; as a result, leukocytes roll on ECs. Neutrophils firmly adhere to ECs by subsequently upregulating CD11b/CD18 [9] and move into sites of inflammation. L-selectin is then crosslinked with L-selectin on other leukocytes, resulting in increased levels of intracellular calcium, p38MAPK activation, and expression of mRNAs for TNF-α and IL-8 [10]. These processes result in aggregation of PMNLs at sites of inflammation and degranulation in response to inflammation.

L-selectin may also be involved in the adhesion between granulocytes and sinusoid matrix ECs in the BM. A continuous increase in the expression of L-selectin and subsequent movement of mature cells from the BM stroma into the sinusoids, along with a gradual decrease in CD34 expression, were observed during the differentiation and maturation of BM blood cells [11]. This process was also affected by similar ligands of adhesion molecules and endogenous proteolytic enzymes [12].

Astragalus polysaccharide (APS) has been shown to significantly increase the numbers of leukocytes, neutrophils, and platelets at 4, 7, 10, and 14 days after chemotherapy [13]. The present study aims to investigate the leukogenic effect of APS, to compare its effect of increasing the numbers of mature granulocytes in the blood with that of G-CSF, and to investigate the underlying mechanism of action.

## Methods

### Ethics statement

The experimental protocols were approved by the Animal Ethical and Welfare Committee of Tianjin Medical University Cancer Institute and Hospital (No. 058, date: 2013.1.7) (Additional file 2). The principles of laboratory animal care and the guidelines of the National Animal Welfare Law of China were followed when applicable.

### Animal and pharmacological investigation on different drugs

Male Wistar rats (weighing 180–230 g) obtained from Charles River Laboratories (China; Certificate No: SCXK (jing) 2012–0001) were housed in cages (200 rats in 40 cages, five rats per cage) placed in temperature-controlled rooms. Rats received water and food *ad libitum* until they were needed for experimentation. The rats were arbitrarily grouped: control group, cyclophosphamide (CTX; Jiangsu Hengrui Medicine Co., Ltd., Jiangsu, China) group, CTX + APS (Tianjin Cinorch Pharmaceutical Co., Ltd., Tianjin, China) group, and CTX + G-CSF (Qilu Pharmaceutical Co., Ltd., Jinan, China) group. Fifty rats were included in each group. Each group was then arbitrarily divided into five subgroups (10 rats per subgroup) to provide samples on different numbers of days after CTX infusion (0, 4, 7, 10, and 14 d). Control animals received 0.4 mL of sterile phosphate-buffered saline (PBS, Gibco BRL Co.Ltd., Grand Island, NY, USA).

Drug doses and schedule of administration were chosen according to the results of previous studies: (1) intraperitoneal injection of 40 mg/kg CTX from day 0 to 1 [14]; (2) intravenous injection of 100 mg/kg APS from day −2 (2 days before CTX injection) to 11 [15]; and (3) subcutaneous injection of 10 μg/kg G-CSF from day 2 to 5 [16].

### Blood and BM sampling

The rats were anesthetized by intraperitoneal injection of chloral hydrate at selected time intervals. Blood samples were drawn from the abdominal artery and placed in vacutainer tubes containing EDTA (BD Vacutainer Systems, Plymouth, UK). The rats were then sacrificed and their femurs were immediately removed. The epiphyses were cut transversely and the BM cells were flushed out with Iscove’s modified Dulbecco’s medium containing 584 mg/LL-glutamine (IMDM; HyClone; China) supplemented with 10 % heat-inactivated fetal calf serum (FCS; Yuanhengjinma Biotechnology Kaifa Co., China) and 100 U/mL lithium heparin. The sternum was removed and extruded for BM smears.

### Fractionation of BM cells and isolation of blood PMNLs

Percoll media (GE Healthcare, Bio-Sciences AB) of different densities were prepared as previously described [17]. A BM cell suspension or whole blood (3 mL) was gently layered on top of a two-layer discontinuous Percoll gradient: 1.093 and 1.077 g/mL (72 % and 60 % Percoll) densities for BM; 1.068 and 1.058 g/mL (51 % and 42 % Percoll) densities for blood. The tubes were then centrifuged at (1000 × *g*, 20 min, room temperature; Multifuge 1R, Thermo Fisher, USA) to separate the rat BM into three fractions. The middle-level cell fraction, which contained PMNLs, was washed twice in 10 mL of PBS (300 × *g*, 10 min, room temperature) to remove the remaining Percoll, and erythrocytes contaminating the pellet were removed using an Optilyse C lysis solution (Beckman Coulter, France). The PMNLs, separated by Percoll, were confirmed as BM neutrophil cell lines at various stages of differentiation by flow cytometry and microscopy using oil lens [18]. We used only samples containing >90 % BM PMNLs (bands and segmented granulocytes) and circulating PMNLs with 95 % cell viability, as determined by trypan blue staining.

### Flow cytometry

Expression of L-selectin, PSGL-1, CD11b/CD18, and ADAM-17 was assessed using a FACScan flow cytometer (Beckman Coulter EPICS-XI, USA). BM bands, segmented cells, and circulating PMNLs were gated in a forward scatter (cell size) *vs.* side scatter (granularity) dot plot. Mature BM granulocyte samples were pretreated with saturating concentrations of phycoerythrobilin (PE)-conjugated hamster anti-rat L-selectin monoclonal antibody (BD Pharmingen, Catalog No. 551398, USA) with mouse anti-rat PSGL-1 monoclonal antibody (KPL-1;Abcam, Catalog No. ab78188, UK), and PE-conjugated mouse anti-rat CD11b/CD18 monoclonal antibody (BD Pharmingen, Catalog No. 562105) with rabbit anti-rat ADAM17 polyclonal antibody (Abcam, Catalog No. ab2051). Fluorescein isothiocyanate (FITC)-labeled goat anti-rabbit IgG and goat anti-mouse IgG (ZSGB-BIO, China) were used as secondary reagents. Negative controls were obtained by omitting monoclonal antibodies. Class-matched irrelevant PE-labeled hamster IgG_2_isotype control (BD Pharmingen, Catalog No. 553965) and FITC-labeled goat anti-mouse IgG were used to evaluate non-specific antibody binding. Data from 100,000 events per sample were acquired. The percentage of fluorescent cells (%F) was used to quantify L-selectin, PSGL-1, and ADAM17 expression. The mean fluorescence intensity (MFI) was used to quantify CD11b/CD18 expression after the neutrophils were gated according to their characteristic forward and side scatter properties.

### Leukocyte countsand morphological analysis

The total leukocyte count in peripheral blood samples was obtained using an automated cell counter (MEK-6318 K, Dobio, Japan). Leukocyte differential counts were obtained for a minimum of 100 cells in blood and BM smears stained with Giemsa. Granulocytic cells were classified into immature granulocytes, mature granulocytes (bands and segmented), eosinophils, and mononuclear cells.

### Preparation of zymosan-activated fetal bovine serum (ZAS)

ZAS was prepared as described previously [19]. In brief, a bovine serum pool with 15 mg/mL of zymosan (Sigma-Aldrich, Switzerland, USA) was incubated at 37 °C for 60 min. The mixture was subsequently incubated at 56 °C for 30 min and cooled. Afterwards, the mixture was centrifuged at 1100 × *g* at 4 °C for 15 min. ZAS was diluted in a 1:5 Hanks balanced salt solution (without calcium and magnesium; Gibco BRL Co. Ltd., USA) and filtered through a 0.22-μm filter (Merck Millipore, Germany).

### Measurement of chemotaxis

A micropore membrane was used to assess the chemotactic ability of mature BM granulocytes and blood PMNLs. Micropore inserts (diameter = 12 mm; pore size = 3 μm; Corning Incorporated, USA) were coated with collagen using a 0.1 % solution of calfskin collagen type I (Gibco, Invitrogen Corporation, Grand Island, USA). The cells were adjusted to 10^6^/mL in PBS. For the treated group and the untreated control group, 200 μL of either PMNL suspension or mature BM granulocytes were added to the upper chambers. For the treated group, 400 μL of ZAS was placed in the lower chamber. For the control group, 400 μL of Hanks balanced salt solution was used. All group samples for the assays were incubated for 90 min at 37 °C in 95 % air and 5 % CO_2_. The migrated PMNLs and mature BM granulocytes were counted using a Burker chamber (Pyser-SGI, UK) at a magnification of 10 × 40. Results are expressed as the percentage of cells that migrated through the membrane. Each assay was performed in duplicate.

### Statistical analysis

Data are expressed as means ± standard deviation (SD). All statistical analyses were performed in SPSS 17.0 (SPSS Inc., Chicago, IL, USA). Data were compared using the Kruskal-Wallis test. Dunn’s multiple contrast hypothesis test was used to compare each treatment group mean with each other group mean. The Mann–Whitney *U* test was used for unpaired observations. A *P* value less than 0.05 was considered significant. The effect of multiple comparisons was corrected for with the Bonferroni method.

## Results

### HE staining of BM

**Fig. 1:**
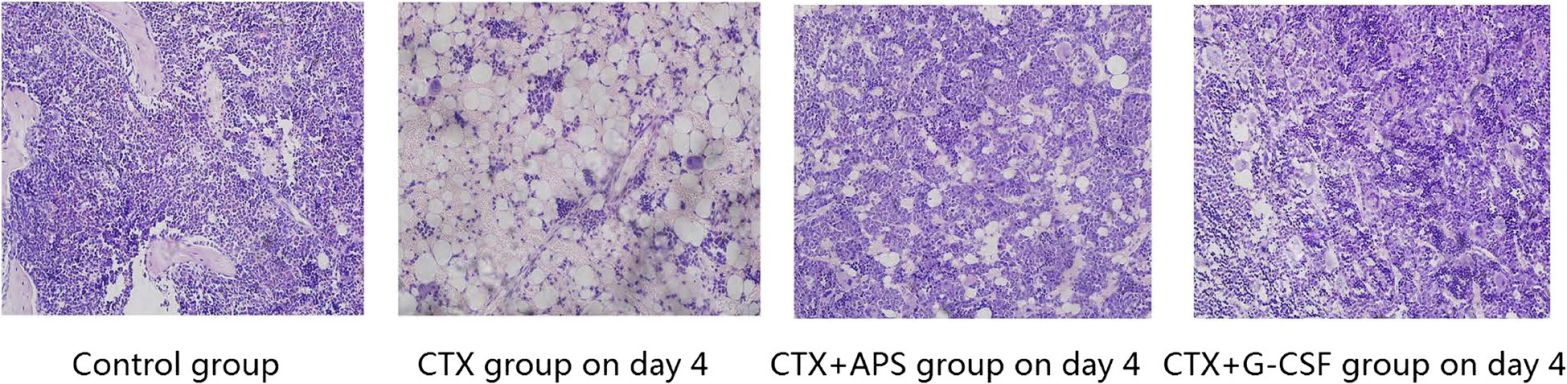
HE staining of BM. BM section of the humerus showed abundant erythroid and myeloid cells, normal differentiation, and visible capillaries in the control group. Four days after CTX chemotherapy, the entire BM cavity was almost filled with fat cells. Erythroid and myeloid cells were significantly decreased, with the latter showing a more obvious decrease. Meanwhile, the structures of microvessels and sinusoids were destroyed. At day 4, erythroid and myeloid proliferation was higher in the APS and G-CSF treatment groups, especially in the G-CSF group, compared with the control group. The structure of capillaries and sinusoids was distorted and fuzzy in the G-CSF group, but clear in the APS group

In the control group, a BM section of the humerus showed abundant erythroid and myeloid cells, normal differentiation, and visible capillaries and sinusoids. Four days after CTX chemotherapy, the entire BM cavity was almost completely filled with adipose cells. Erythroid and myeloid cells were significantly decreased in number (this was more evident for myeloid cells), whereas the structures of microvessels and sinusoids had been destroyed. On day 4, the proliferation of erythroid and myeloid cells was higher in the APS and G-CSF treatment groups than in the CTX group, particularly in the G-CSF group; the latter had capillaries and sinusoids showing distorted and fuzzy structures, which were not seen in the APS group (Fig. 1). On day 14, BM proliferation (erythroid and myeloid cells), was active in all three CTX treated groups without significant difference (Additional file 3).

### Peripheral blood leukocyte and neutrophil count

**Fig. 2:**
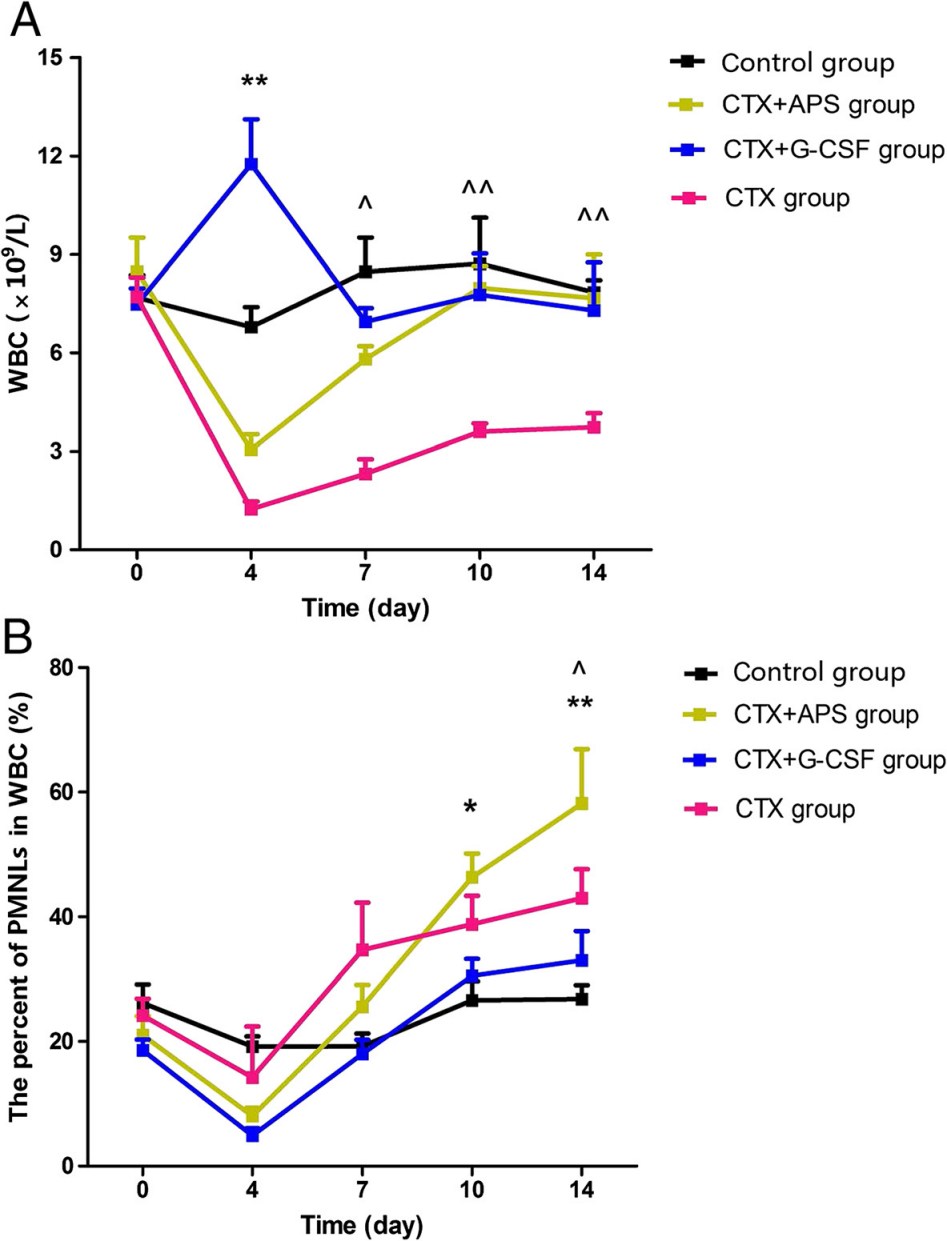
Peripheral blood leukocyte and neutrophil count. Total leukocyte count of the peripheral blood samples was obtained using an automated cell counter. Leukocyte differential count was carried out on a minimum of 100 cells. **a** Peripheral blood leukocyte and neutrophil count after drug administration at different time points. In the G-CSF group, the number of total white blood cells significantly increased on day 4 and then gradually decreased thereafter. In the APS group, total leukocyte count gradually increased. No statistical difference was observed between the APS and G-CSF groups after 7 days (*P* > 0.05). **b** With APS and G-CSF administration, the proportion of peripheral blood PMNLs in total white blood cells started to increase, and a significant increase was observed in the APS group on day 14 (*vs.* the CTX group and G-CSF groups, *P* < 0.05). However, the percentage of PMNLs in total white blood cells in the G-CSF group was slightly lower than that of the CTX group at each time point (*P* > 0.05). Data were expressed as means ± SD. Comparison of CTX + APS and CTX + G-CSF groups showed a significant difference (**:*P* < 0.01, *: *P* < 0.05). Comparison of CTX + APS group and CTX group showed a significant difference (^ ^: *P* < 0.01, ^: *P* < 0.05)

The proportion of peripheral blood PMNLs among total white blood cells of the CTX group was not significantly lower than that in the control group on day 4 after chemotherapy (*P* = 0.595). On day 7, the percentage of PMNLs in the CTX group began to increase. After APS and G-CSF administration, the proportion of peripheral blood PMNLs among total white blood cells became similar to that for the CTX group at each time point, and a significant increase was observed in the APS group on day 14 compared with other groups (*P* = 0.001 *vs.* control group, *P* = 0.006 *vs.* G-CSF group, *P* = 0.047 *vs.* CTX group). The percentage of PMNLs in the G-CSF group was not significantly lower than that in the CTX group at each time point (*P* = 0.108 on day 4, *P* = 0.053 on day 7, *P* = 0.068 on day 10, *P* = 0.207 on day 14; Fig. 2). On day 4, the white blood cell count in the G-CSF group was the highest (almost 12 × 10^9^/L) among all groups, but the percentage of PMNLs among white blood cells was only 5 %.

### Expression of L-selectin, PSGL-1, ADAM17, and CD11b/CD18

On day 4, the positive expression rate (F%) of L-selectin in mature BM granulocytes in the CTX group was 59.10 ± 8.45 %, which was higher than that in the control group (*P* < 0.001). F% decreased on days 7, 10, and 14. A significant difference was observed on days 7 and 14 (*P* < 0.001 on day 7 and *P* = 0.043 on day 14*vs.* control group), whereas F% in the APS group gradually increased from day 4 to 14 after chemotherapy and reached 49.53 ± 8.92 % on day 14. F% of L-selectin in the G-CSF group was the highest on day 4 (48.60 ± 3.08 %) and gradually decreased to 28.67 ± 9.10 % on day 14. In contrast, F% of L-selectin in the APS group was higher than that of the G-CSF group after 7 days (*P* = 0.005 on day 7, *P* < 0.001 on day 10, *P* < 0.001 on day 14). On day 4, F% of L-selectin in the two treatment groups was significantly lower than that in the CTX group (*P* < 0.001 *vs.* APS group, *P* = 0.001 *vs.* G-CSF group), but significantly higher than that in the CTX group after 7 days (*P* < 0.001 on day 7, *P* < 0.001 on day 10, *P* < 0.001 on day 14 *vs.* APS group; *P* < 0.001 on day 7, *P* = 0.001 on day 10, *P* = 0.003 on day 14 *vs.* G-CSF group).

**Fig. 3:**
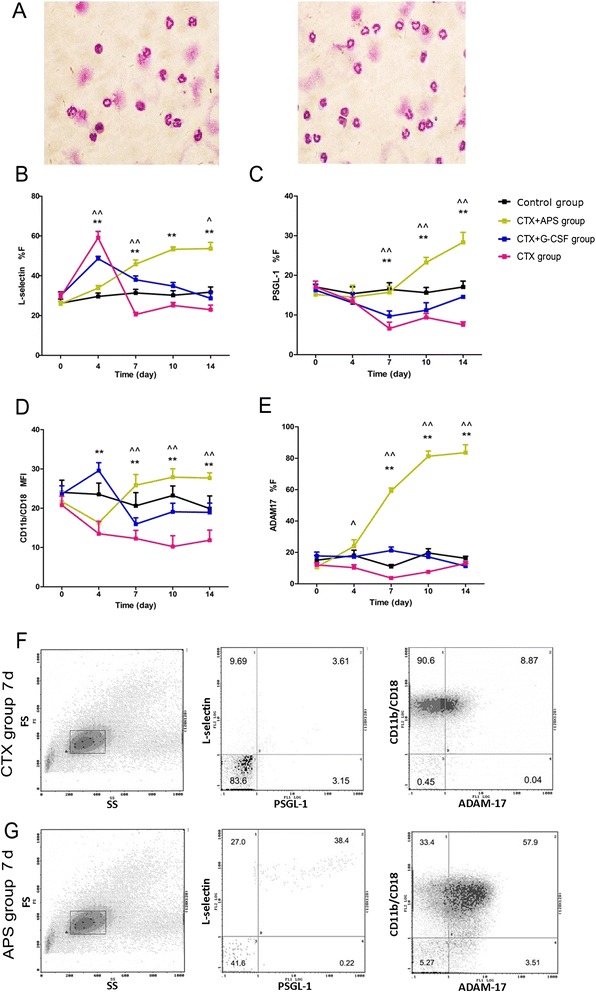
Expression of L-selectin, PSGL-1, ADAM17 and CD11b/CD18. **a** Light microscopy of the gated cells obtained after sorting the corresponding fractions (original magnification, 600 ×). **b** %F of L-selectin expression on the cell surface of mature BM granulocytes in each group. **c** %F of PSGL-1 expression on the cell surface of mature BM granulocytes in each group. **d** %F of ADAM-17 expression on the cell surface of mature BM granulocytes in each group. **e** MFI of CD11b/CD18 expression on the cell surface of mature BM granulocytes in each group. **f** and **g** Flow cytometry analysis on mature BM granulocytes after density gradient centrifugation: forward scatter (FS) *vs.* side scatter (SS) dot plot, and scatter plot of protein expression in CTX and APS groups. Data were expressed as means ± SD. Comparison of CTX + APS group and CTX + G-CSF group showed a significant difference (**:*P* < 0.01, *: *P* < 0.05). Comparison of CTX + APS group and CTX group showed a significant difference (^ ^: *P* < 0.01, ^: *P* < 0.05)

F% of PSGL-1 in the mature BM granulocytes after CTX treatment decreased gradually on days 4–14. No significant change in F% was observed in the APS group on days 4 and 7 (*P* = 0.776 on day 4, *P* = 0.788 on day 7*vs.* control group); however, F% gradually increased from day 10 to day 14 (23.25 ± 8.78 % and 28.33 ± 6.63 %, respectively; *P* < 0.001 on day 10, *P* < 0.001 on day 14*vs.* control group). On day 7, F% of PSGL-1 in the APS group was significantly higher than that in the CTX group (*P* < 0.001); however, no significant difference in F% was observed between the G-CSF and CTX groups before day 14 (*P* = 0.415 on day 4, *P* = 0.802 on day 7, *P* = 0.889 on day 10). F% gradually decreased after G-CSF was injected, and the lowest value was reached on day 7 (9.09 ± 3.87 %). On days 7, 10, and 14, F% of PSGL-1 in the APS group was higher than that in the G-CSF group (*P* = 0.038 on day 7, *P* = 0.001 on day 10, *P* < 0.001 on day 14; Fig. 3).

F% of ADAM17 in mature BM granulocytes in the CTX group on days 4 to 14 was insignificantly lower than that in the control group (*P* = 0.176 on day 4, *P* = 0.204 on day 7, *P* = 0.101 on day 10, *P* = 0.368 on day 14). At each time point, the APS group showed higher F% than did the control, CTX, and G-CSF groups (*P* = 0.007 on day 4, *P* < 0.001 on day 7, *P* < 0.001 on day 10, *P* < 0.001 on day 14 *vs.* control group; *P* = 0.003 on day 4, *P* < 0.001 on day 7, *P* < 0.001 on day 10, *P* < 0.001 on day 14 *vs.* CTX group; *P* = 0.006 on day 4, *P* < 0.001 on day 7, *P* < 0.001 on day 10, *P* < 0.001 on day 14 *vs.* G-CSF group), with the maximum F% of 83.52 ± 12.36 % obtained 14 days after chemotherapy. F% of ADAM17 in the G-CSF group did not change significantly at each time point (*P* = 0.892 on day 4, *P* = 0.574 on day 7, *P* = 0.727 on day 10, *P* = 0.822 on day 14 *vs.* control group; *P* = 0.425 on day 4, *P* = 0.168 on day 7, *P* = 0.062 on day 10, *P* = 0.916 on day 14*vs.* CTX group; Fig. 3).

MFI of CD11b/CD18 on mature BM granulocytes significantly decreased after CTX treatment, and was further reduced by 50 % on days 10 and 14. The minimum MFI (15.98 ± 8.16) in the APS group was obtained on day 4; the MFI was increased significantly on days 7, 10, and 14 (*P* = 0.154 on day 7, *P* = 0.169 on day 10, *P* = 0.052 on day 14*vs.* control group, *P* = 0.028 on day 7, *P* < 0.001 on day 10, *P* < 0.001 on day 14*vs.* CTX group). The maximum MFI (29.56 ± 5.12) was obtained in the G-CSF group on day 4 (*P* = 0.136*vs.* control group). MFI in the G-CSF group was significantly decreased on days 7, 10, and 14 compared with that in the APS group (*P* < 0.001 on day 7, *P* < 0.001 on day 10, *P* < 0.001 on day 14). MFI in the G-CSF group was lower than that in the control group (*P* > 0.05) but insignificantly higher than that in the CTX group (*P* > 0.05) (Fig. 3).

### Differentiation of BM granulocytes

**Fig. 4:**
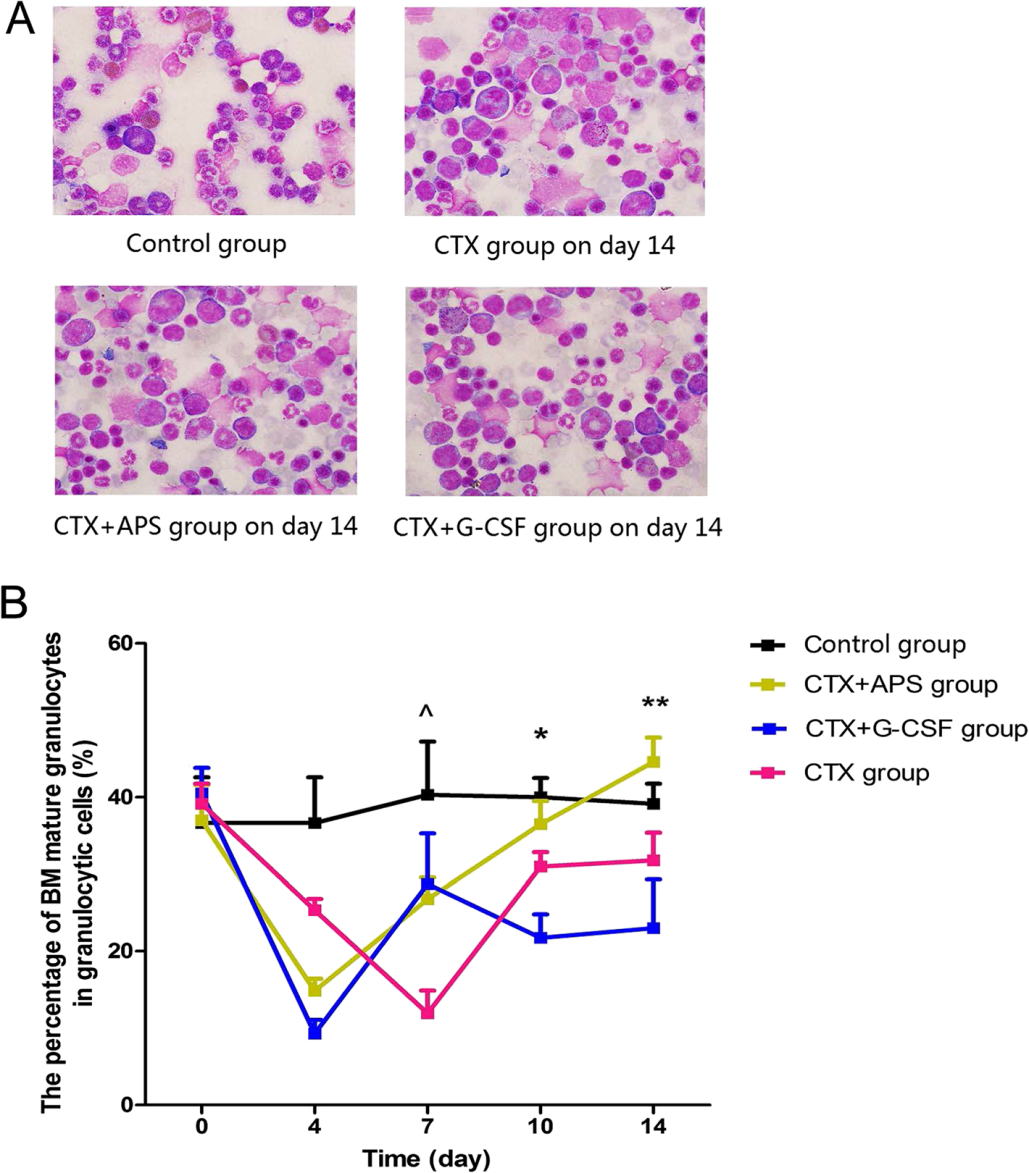
Differentiation of BM granulocytes. BM smears collected from the sternum at different time points were used. We determined the proportion of BM bands and segmented cells in the total number of neutrophils, and used a microscope to identify the degree of differentiation of BM neutrophils. **a** BM smears observed under oil microscopy (600×) showed granulocyte differentiation on day 14. **b** Leukocyte differential counts were carried out on a minimum of 500 cells on BM smears stained with Giemsa. Cells of the granulocytic lineage were classified as immature granulocytes (myeloblasts, promyelocytes, myelocytes, and metamyelocytes), mature granulocytes (bands and segmented), eosinophils, and mononuclear cells. Data were expressed as means ± SD. Comparison of CTX + APS group and CTX + G-CSF group showed a significant difference (**:*P* < 0.01, *: *P* < 0.05). Comparison of CTX + APS group and CTX group showed a significant difference (^ ^: *P* < 0.01, ^:*P* < 0.05)

Using the BM smears collected from the sternum, we counted the proportion of bands and segmented cells in the total number of neutrophils under a microscope to identify the degree of BM neutrophil differentiation. The lowest percentage of BM bands and segmented cells in the CTX group was observed on days 4 and 7 (24.70 ± 5.77 % and 19.45 ± 7.23 %, respectively), but this proportion subsequently increased on days 10 and 14 (*P* = 0.032 on day 10, *P* = 0.127 on day 14*vs.* control group). After day 4, the percentages in the APS and G-CSF groups gradually increased as well, while on day 7, the percentage of BM bands and segmented cells was higher than that in the CTX group (*P* = 0.030 *vs.* APS group, *P* = 0.016 *vs.* G-CSF group), although this result was not statistically different from the CTX group results on days 10 and 14. The percentage in the APS group significantly increased and reached normal levels on days 10 and 14 (*P* = 0.547 on day 10 and *P* = 0.431 on day 14*vs.* control group; *P* = 0.015 on day 10 and *P* = 0.007 on day 14*vs.* G-CSF group; Fig. 4).

### Chemotactic ability of mature BM granulocytes and peripheral blood PMNLs

**Fig. 5:**
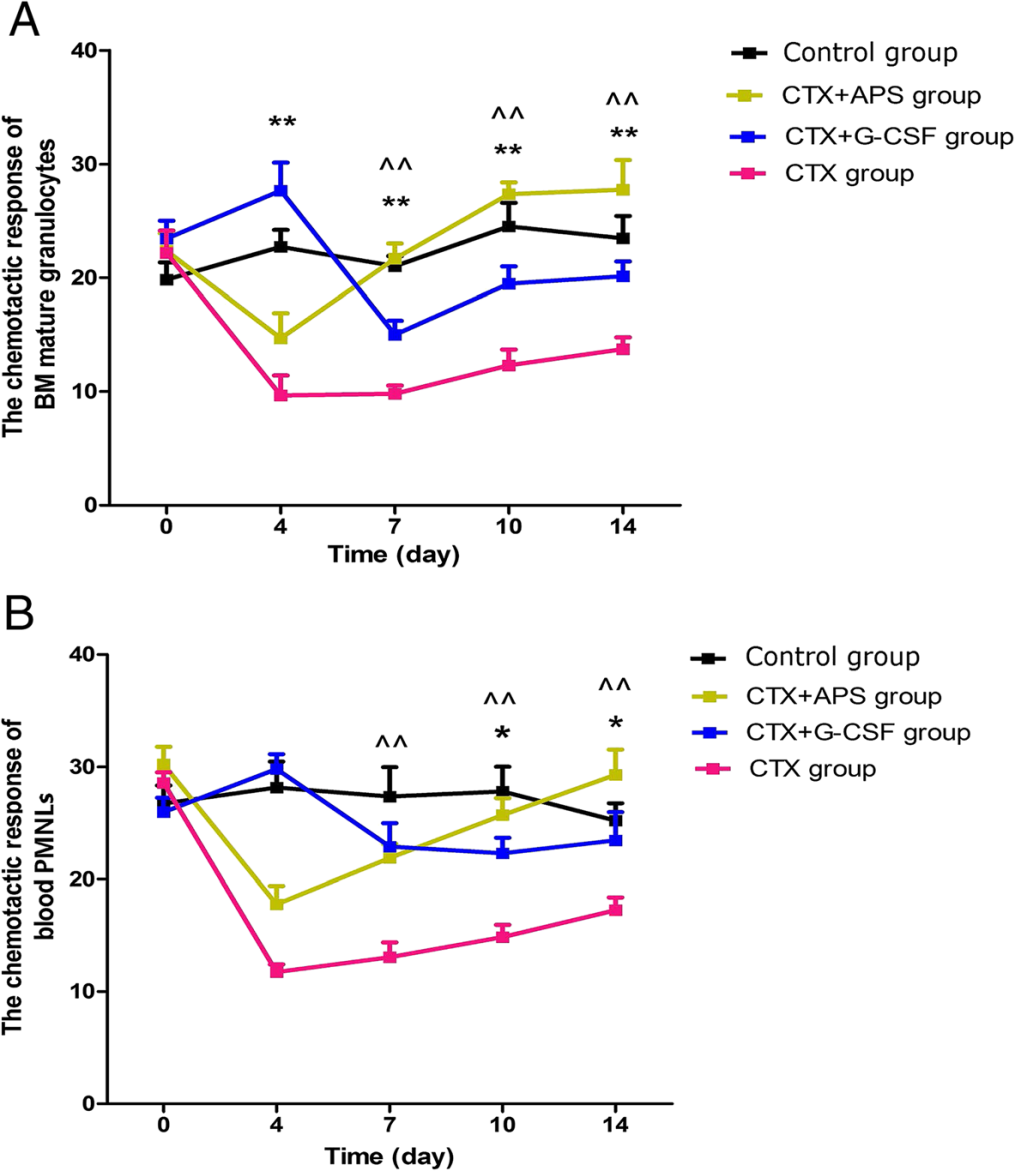
Chemotactic ability of mature BM granulocytes and peripheral blood PMNLs. A micropore membrane was used to assess the chemotactic ability of mature BM granulocytes (**a**) and blood PMNLs (**b**). The chemotactic ability of mature BM granulocytes and peripheral blood PMNLs significantly decreased at each time point. In the CTX group, the minimum value was obtained on day 4 (*P* < 0.001 *vs.* control group). At each time point thereafter, the values were significantly lower than those of the other treatment groups, and the value of the CTX group was only 50 % of control group on day 14. In the APS group, the minimum value obtained on day 4, before gradually increasing on days 7, 10, and 14. The chemotactic ability of both mature BM granulocytes and peripheral blood PMNLs of the APS group were higher than those of the CTX group (*P* < 0.01), and the G-CSF group, especially 10 and 14 days after chemotherapy. The maximum value obtained in the CTX group on day 4 was not significantly different with that of the control group. The decrease in values observed on day 7, 10, and 14 was not significant. On day 10 and 14, the chemotactic ability in the G-CSF group was lower than that of the control and APS groups (*P* < 0.05). Compared with the CTX group, the chemotactic ability of mature BM granulocytes and peripheral blood PMNLs in APS group improved significantly after 7 days. Data were expressed as means ± SD. Comparison of CTX + APS group and CTX + G-CSF group showed a significant difference (**:*P* < 0.01, *: *P* < 0.05). Comparison of CTX + APS group and CTX group showed a significant difference (^ ^: *P* < 0.01, ^: *P* < 0.05)

The chemotactic ability of mature BM granulocytes and peripheral blood PMNLs in the CTX group was significantly decreased compared with that of cells in the control group at each time point. In the CTX group, the minimum percentage value was obtained on day 4 (*P* < 0.001*vs.* control group). The value for the CTX group was approximately 50 % of that for the control group on day 14. In the APS group, the minimum percentage value was obtained on day 4, before gradually increased on days 7, 10, and 14. The chemotactic ability of mature BM granulocytes and peripheral blood PMNLs was higher in the APS group than in the CTX group (*P* < 0.001 on days 7, 10 and 14) and the G-CSF group (*P* < 0.001 on day 7, *P* = 0.001 on day 10 and *P* = 0.005 on day 14), particularly on days 10 and 14 after chemotherapy. In the CTX group, the maximum value was obtained on day 4, but this result was not significantly different from that in the control group. A decrease was observed on days 7, 10, and 14, but the decrease was not significant. On days 10 and 14, the chemotactic ability in the G-CSF group was lower than that in the control and APS groups (*P* = 0.028 on day 10 and *P* = 0.017 on day 14 *vs.* control group; *P* = 0.001 on day 10 and *P* = 0.005 on day 14 *vs.* APS group), and mature BM granulocytes and peripheral blood PMNLs in the APS group showed significant improvements in such capabilities after 7 days relative to those in the CTX group (Fig. 5).

## Discussion

### Design of treatment protocols

The administration of 10 μg/kg G-CSF from day 2 to day 5 after chemotherapy based on a previous report [16] and the results of our pilot experiment could enable the maximal stimulation of proliferation of neutrophils to be achieved, leading to severe leakage from vessels, edema, and even the death of several rats [15]. APS was given in different ways in our pilot studies to obtain the maximal effect; ‘pre-chemotherapeutic administration’ and maintenance for 11 days after chemotherapy were determined to be the best protocols to increase neutrophils. The most suitable protocols for the two drugs were chosen to compare their maximal effects and to ensure safety in animals.

### Effects on adhesion/release-related protein expression in mature BM granulocytes

Frenette and Weiss [20] confirmed that PMNLs expressing L-selectin can be preferentially released from the BM into the peripheral blood. ADAM17 is a major proteolytic enzyme that hydrolyzes the extracellular and juxtamembrane region of L-selectin [21]. Our results showed that CTX could reduce expression of L-selectin, PSGL-1, CD11b/CD18, and ADAM17 from day 7–14 by interfering with initial binding, stable adhesion between granulocytes and sinusoidal endothelial cells, and smooth PMNL release. G-CSF could reduce this interference to a certain extent, but the effect was short lived; contrastingly, APS could significantly increase the expression levels of key proteins involved in granulocyte adhesion and release for a longer time (maximum of 7 days to 14 days). To our knowledge, our study is the first to elucidate that APS could relieve chemotherapy-induced impairment on key factors related to adhesion and release of PMNLs.

### Effects on microvessel and sinusoid structures in BM

Chemotherapeutic agents could significantly reduce hematopoietic islets, resulting in severe suppression in bone marrow hematopoiesis [22]. However, the reasons for chemotherapy-induced anemia should be more than just inhibition of proliferation of hematopoietic cells alone. Our findings revealed that CTX resulted in a fuzzy structure of microvessels and sinusoids in BM 4 days after chemotherapy. This damage in a hematopoietic microenvironment could impair the environment for proliferation and differentiation of hematopoietic cells, and also interfere with the adhesion and release of granulocytes. G-CSF could not restore the structure of microvessels and sinusoidal ECs. After APS treatment, the structures of BM microvessels and sinusoids became clear and organized, and adhesion and release of mature BM granulocytes were promoted. Given the enhancement of the immunocytotoxic function of APS against malignancies [23], we believe that its ‘protection’ of the bone marrow stroma could hardly protect tumors from chemotherapy.

### Effects on the differentiation and release of granulocytes in BM

The BM section of the humerus showed that the BM cavity was filled with adipose cells 4 days after chemotherapy and erythroid and myeloid cell counts significantly decreased. Xue *et al.* [24, 25] found a severe defect in BM by loosing hematopoietic cells in VEGF tumor-bearing mice probably due to possibility for VEGF to mobilize BM hematopoietic stem cells to peripheral tissues and organs. After APS and G-CSF administration, the proliferation of myeloid cells was observed. In the G-CSF group, the ratio of mature BM granulocytes to the total neutrophils was lower than that of the CTX group, suggesting that G-CSF remarkably stimulated the proliferation of naive hematopoietic cells [26]. Only 5 % of PMNLs were found 4 days after chemotherapy, when the highest levels of WBC were found in G-CSF group, suggesting that G-CSF prompts non-PMNLs releasing from bone marrow to peripheral blood. Janowska-Wieczorek *et al.* [27] demonstrated that G-CSF-induced the release of gelatinase B, which has a major function in the migration of BM early-stage myeloid cells into the blood. The total white blood cells reached normal level as induced by APS at day 14, while the proportion of mature BM granulocytes to the total neutrophils reached normal levels at day 10 and 14 in the APS group. Above results confirmed that APS promoted myeloid differentiation and maturation, and reduced mobilization of naïve BM hematopoietic cells to peripheral tissues, which yield a question that ought to be explored and might be the possibility for APS to restore BM hematopoiesis in patient with advanced cancer. APS augmented the expression of signalling molecules, releasing PMNLs after chemotherapy. This result is consistent with what is observed in leukemia cells with lower L-selectin expression, showing impaired transendothelial migration *in vitro* [28].

### Effects on the chemotactic ability

To assess the chemotactic ability of PMNLs, we measured the ability of mature BM granulocytes and circulating PMNLs to pass through collagen-coated membranes to ZAS. The chemotactic ability of BM and PMNLs in the CTX group markedly decreased to 50 % of that in the control group at 14 d after chemotherapy. G-CSF could increase the chemotactic ability of PMNLs until day 7 after chemotherapy, but APS consistently increased this capability of PMNLs more effectively than G-CSF did. This result mightbe attributable to the upregulation of key proteins, such as TNF-α and IL-8, in the L-selectin signalling pathway in PNMLs by APS.

The activation of human granulocytes *via* cross-linking between L-selectin and its antibody can increase intracellular Ca^2+^ concentration [29], production and phosphorylation of superoxides [30], and activation of key signalling proteins [31]. Cross-linking of L-selectin and its ligand as sulphated galactocerebroside increased the expression of TNF-α and IL-8 [10], causing granulocytes to penetrate the layer of human umbilical vein ECs. The inability of G-CSF to increase the chemotactic ability was probably due to its inability to restore the key proteins in the L-selectin signalling pathway. High expression of CD11b/CD18 was observed after the activation of granulocytes by cross-linking of L-selectin, which is closely related to the chemotactic ability of granulocytes in addition to mediating the stable adhesion of granulocytes [30].

## Conclusions

APS promoted differentiation and chemotactic ability of BM granulocytes, involving the L-selectin signalling pathway.

## Additional files

Additional file 1:
**HE staining of BM of a lung cancer patient (45 years old, male, 7 days after his 3rd cycle of chemotherapy) shows active myeloproliferation.**
Additional file 2:
**The Certification of the approval of The Laboratory Animal Ethic Committee.**
Additional file 3:
**HE staining of BM.** BM section of the humerus showed abundant erythroid and myeloid cells, normal differentiation, and visible capillaries in the control group. Four days after CTX chemotherapy, the entire BM cavity was almost filled with fat cells. Erythroid and myeloid cells were significantly decreased, with the latter showing a more obvious decrease. Meanwhile, the structures of microvessels and sinusoids were destroyed. At day 4, erythroid and myeloid proliferation was higher in the APS and G-CSF treatment groups, especially in the G-CSF group, compared with the control group. The structure of capillaries and sinusoids was distorted and fuzzy in the G-CSF group, but clear in the APS group. On day 14, BM proliferation was active in all three CTX treated groups without significant difference.

## Abbreviations

APS: Astragalus polysaccharide
BM: Bone marrow
CTX: Cyclophosphamide
ECs: Endothelial cells
FITC: Fluorescein isothiocyanate
FS: Forward scatter
G-CSF: Granulocyte colony-stimulating factor
L-selectin: Leukocyte selectin
MFI: Mean fluorescence intensity
MMP-9: Matrix metalloproteinase-9
PBS: Phosphate-buffered saline
PE: Phycoerythrobilin
PMNLs: Polymorphonuclear leukocytes
PSGL-1: P-selectin glycoprotein ligand-1
SD: Standard deviation
SS: Side scatter
ZAS: Zymosan-activated fetal bovine serum
