# Supplementary material for: Astragalus polysaccharide promotes the release of mature granulocytes through the L-selectin signaling pathway
Source: Chin Med. 2015 Jul 3;10:17. doi: 10.1186/s13020-015-0043-z (PMC4497426; doi:10.1186/s13020-015-0043-z)
Supplement: Additional file 1: — HE staining of BM of a lung cancer patient (45 years old, male, 7 days after his 3rd cycle of chemotherapy) shows active myeloproliferation. [file 13020_2015_43_MOESM1_ESM.pdf]

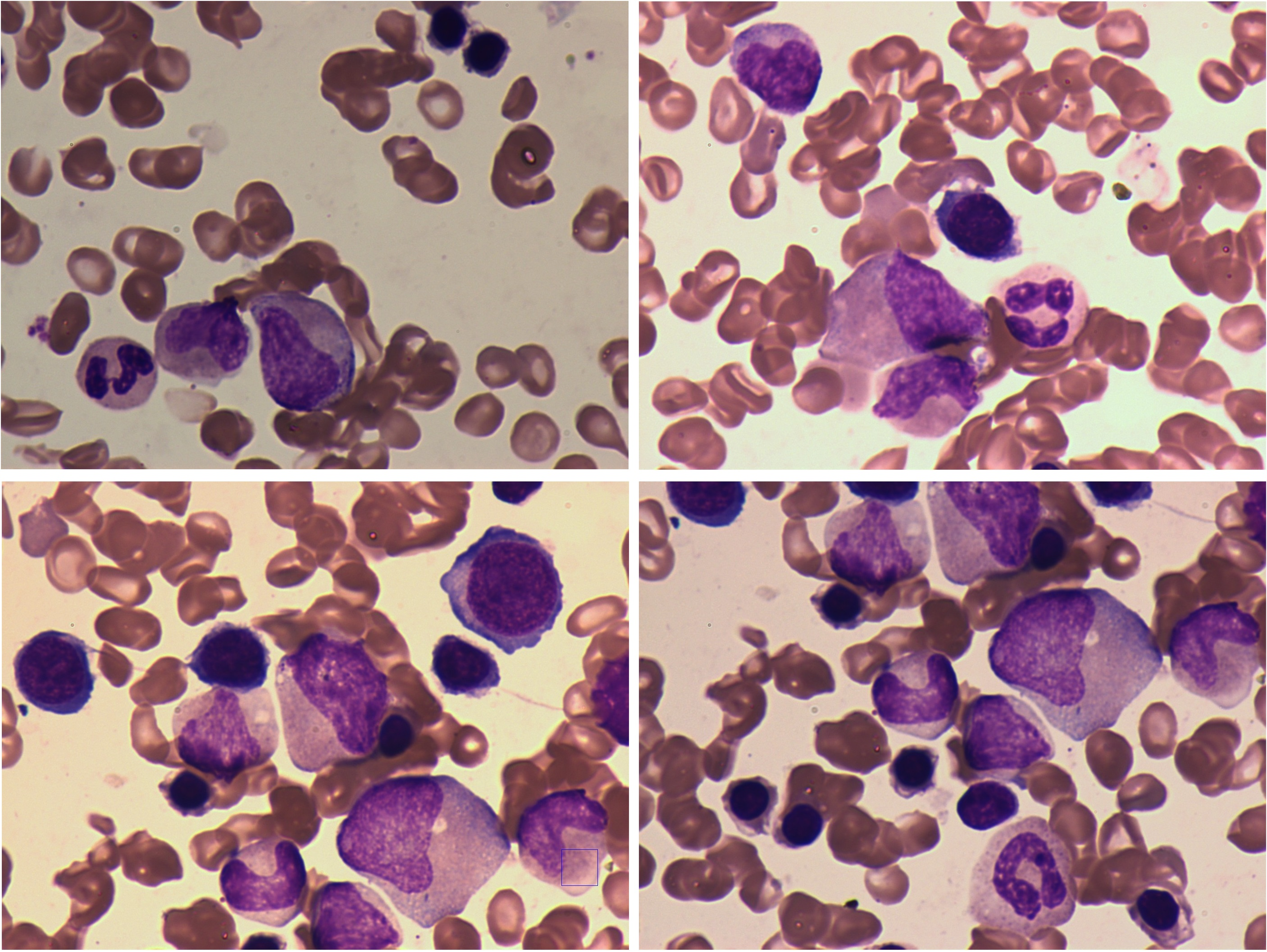

Additional file 01. HE staining of BM of a lung cancer patient (45 years old, male, 7 days after his 3rd cycle of chemotherapy) shows active myeloproliferation .
