# Supplementary material for: Astragalus polysaccharide promotes the release of mature granulocytes through the L-selectin signaling pathway
Source: Chin Med. 2015 Jul 3;10:17. doi: 10.1186/s13020-015-0043-z (PMC4497426; doi:10.1186/s13020-015-0043-z)
Supplement: Additional file 2: — The Certification of the approval of The Laboratory Animal Ethic Committee. [file 13020_2015_43_MOESM2_ESM.pdf]

实验动物伦理审批同意书

The Certification of the approval of The Laboratory Animal Ethic Committee

审批编号 Approved No.: 058

科室<br>Name of department	肺部肿瘤内科<br>Department of Thoracic Medical Oncology	课题负责人<br>Principal investigator (PI)	李凯<br>Kai Li
实验名称<br>Name of experiment	L-选择素对骨髓粒细胞释放影响的研究<br>Study on effect of L-selectin on granulocyte releasing from bone marrow		
项目类别<br>Category of Project	天津市应用基础与前沿技术研究计划<br>Tianjin Research Program of Application Foundation and Advanced Technology	申请日期<br>Application Date	2013.1.7<br>2013.1.7
动物种系<br>Species or Strains	Wistar 大鼠<br>Wistar rats	动物数量<br>Quantity	200<br>200
实验动物来源<br>Source of laboratory animal	北京维通利华实验动物技术有限公司<br>Charles River Laboratories		
质量合格证编号<br>Number of qualification	SCXK（京）2012/0001<br>SCXK(jing) 2012/0001		
饲养设施条件<br>Condition of the housing facilities	☑ 屏障设施 Barrier housing facility<br>☐ 普通设施 Ordinary housing facility		
许可证编号<br>Number of permit	SYXK（津）2012-0005	有效期：2012.9.14-2017.9.13<br>Term of validity:2012.9.14-2017.9.13	
许可证发放机构<br>Permitting organization	☑天津市科学技术委员会<br>Tianjin Municipal Science and Technology Commission		
审查意见<br>Result of inspection	☑符合动物伦理要求，可以进行实验 Agree		
设施负责人意见<br>Chief Facility Officer	☑同意 Agree<br>☐不同意 Disagree		
备注<br>Supplement	天津医科大学肿瘤医院<br>实验动物伦理委员会<br>（签章）<br>(Stamp of The Animal Ethical and Welfare Committee of Tianjin Medical University Cancer Institute and Hospital)<br>日期：2013 年 1 月 7 日<br>Date:		
