# Supplementary material for: Astragalus polysaccharide promotes the release of mature granulocytes through the L-selectin signaling pathway
Source: Chin Med. 2015 Jul 3;10:17. doi: 10.1186/s13020-015-0043-z (PMC4497426; doi:10.1186/s13020-015-0043-z)
Supplement: Additional file 3: — HE staining of BM. BM section of the humerus showed abundant erythroid and myeloid cells, normal differentiation, and visible capillaries in the control group. Four days after CTX chemotherapy, the entire BM cavity was almost filled with fat cells. Erythroid and myeloid cells were significantly decreased, with the latter showing a more obvious decrease. Meanwhile, the structures of microvessels and sinusoids were destroyed. At day 4, erythroid and myeloid proliferation was higher in the APS and G-CSF treatment groups, especially in the G-CSF group, compared with the control group. The structure of capillaries and sinusoids was distorted and fuzzy in the G-CSF group, but clear in the APS group. On day 14, BM proliferation was active in all three CTX treated groups without significant difference. [file 13020_2015_43_MOESM3_ESM.pdf]

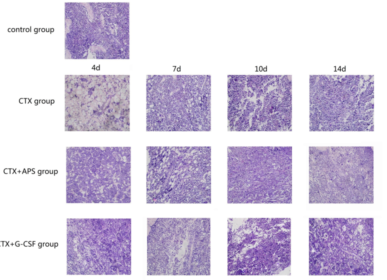


Additional file 03. HE staining of BM.
BM section of the humerus showed abundant erythroid and myeloid cells, normal differentiation, and visible capillaries in the control group. Four days after CTX chemotherapy, the entire BM cavity was almost filled with fat cells. Erythroid and myeloid cells were significantly decreased, with the latter showing a more obvious decrease. Meanwhile, the structures of microvessels and sinusoids were destroyed. At day 4, erythroid and myeloid proliferation was higher in the APS and G-CSF treatment groups, especially in the G-CSF group, compared with the control group. The structure of capillaries and sinusoids was distorted and fuzzy in the G-CSF group, but clear in the APS group. On day 14, BM proliferation was active in all three CTX treated groups without significant difference.
